# Time-scale analysis of the long-term variability of human gut microbiota characteristics in Chinese individuals

**DOI:** 10.1038/s42003-022-04359-9

**Published:** 2022-12-23

**Authors:** Na Han, Tingting Zhang, Yujun Qiang, Xianhui Peng, Xiuwen Li, Wen Zhang

**Affiliations:** State Key Laboratory for Infectious Disease Prevention and Control, National Institute for Communicable Disease Control and Prevention, Chinese Center for Disease Control and Prevention, Beijing, 102206 China

**Keywords:** Next-generation sequencing, Microbiome

## Abstract

Studying the dynamics and stability of the human gut microbiota over time is important for exploring their relationship with human health and developing treatment strategies for putative microbiome-related ailments. Here, we collected stool samples from seven healthy Chinese subjects at 1-month intervals between 2016 and 2020. Sequencing and bioinformatics analyses revealed that the bacteria in the collected fecal samples fluctuated over time, and the extent of these changes increased over time. Further, the average shared proportion value obtained using Sourcetracker2 was 63.5% for samples collected from the same individual in the preceding month, and over a 3-year period, this value decreased to 40.7%. Furthermore, the proportion of different bacteria in the gut microbiota of the different subjects fluctuated to varying degrees. Therefore, our results suggested that it is important to consider the effect of time on gut microbiota composition when it is used to evaluate health. Our study opens up a new field of microbiota research, considering not just the instantaneous microbiota, but also the change of the gut microbiota over time.

## Abstract (web summary)

A study for human gut microbiota composition in 7 healthy Chinese adult subjects across several years provides a valuable perspective on the dynamics of microbiota composition over time.

## Introduction

The human microbiota is essential to the health of the host and can be affected by many features^1,2^. Use of antimicrobial drugs are known to have pronounced effects on the human gut microbiota^3^. Further, it has been observed that gut microbiota disorders can be symptomatic or indicative of a predisposition to several diseases, such as allergies^4^, obesity^5^, diabetes^6^, and even mental illness^7^, and also appear to affect cancer immunotherapy^8,9^. Furthermore, even in healthy adults, gut microbiome characteristics vary with respect to geographical location among populations^10^. For instance, adults originating from the Amazonas of Venezuela, rural Malawi, and US metropolitan areas have different gut microbiota characteristics^11^. It has also been observed that individual differences in gut microbiota composition are closely related to geographical location^12,13^. However, the variation of gut microbiota over time has not yet been sufficiently investigated. A previous study that was focused on changes in gut microbiota composition over time showed that the intermediate-term variation (6 months) of gut microbiota characteristics corresponding to samples collected from the same individual was greater than that observed over the short-term (24 h)^14^. A study lasting 3 months in 2014 also found evidence that the shared proportion of phylotypes in gut microbiota of a single individual at two time points decreased as the interval between the time points increased^15^. Further, another study revealed greater similarity between strains over shorter time intervals than longer time intervals^16^. However, a quantitative and in-depth understanding of the changes in the gut microbiotaover time across years using sequencing technology, as well as their relationship with other factors is still lacking. Therefore, more studies are needed on the medium-term changes to the gut microbiota of healthy individuals.

To this end, in 2016, the Chinese Center for Disease Control and Prevention commenced a study that involved the investigation of the gut microbiome of healthy Chinese individuals. As a part of this project, we conducted a time-scale analysis on fecal samples from seven healthy Chinese subjects, who agreed to participate in an epidemiological survey each month within the 2016–2020 period. Our findings quantitatively clarified gut microbiota changes in healthy Chinese subjects over time and suggested that it is important to consider the effects of time when using gut microbiota to evaluate human health or diagnostic outcomes.

## Results

### Fluctuation of bacterial abundance in individuals with time

**Fig. 1:**
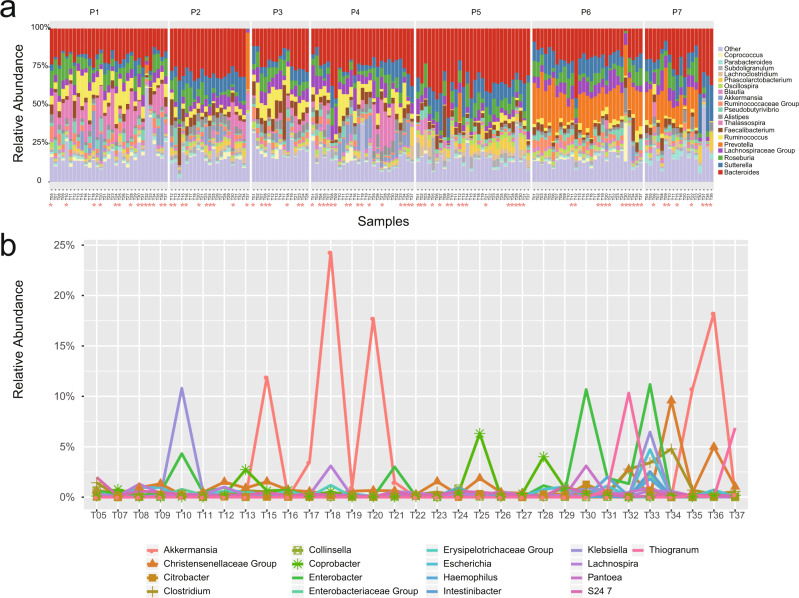
Bacterial abundance in individuals with time. **a** Percentage of different bacterial genera in fecal samples from seven participants at different time points between 2016 and 2020. Only taxa with abundance ≥ 0.1% are shown. “T” on the *x*-axis represents the sample time point and the detailed information for these time points is listed in Supplementary Data 1. * on the x axis represents a short time bloom of particular microbes at this time point at the genus level. **b** The line charts for the short-term blooms of particular microbes in samples from individual P1.T, time point (Supplementary Data 1).

After analyzing 171 fecal samples collected from the volunteers at 37 time points and identifying the microbiota to phylum/genus/species/OTU/ASV level, we observed that the percentage of bacteria in different samples from the same volunteer were not stable over time (Fig. 1).

We observed changes in the bacterial community at different time points and noticed that these changes were not directional, but rather represented fluctuation. After an increase at a certain time point, the abundance returned to normal. If the abundance of a particular genus in one sample is >5 times the average abundance of the genus from the same individual, then we label that genus as being in a short-term bloom in that sample. The short-term blooms of particular microbes in the community occurred in 22 samples (12.9%) from 7 individuals at the phylum level (Supplementary Fig. 1a). The same short-term blooms pattern also happens at the genus level (Fig. 1a), and species level (Supplementary Fig. 1b). At the genus level, we identified a total of 83 samples (48.5%) with short-term blooms covering 45 bacterial genera (Fig. 1a). For example, *Clostridium* was detected in all samples from participant P1. Its abundance increased at time points T33 and T34, followed by a decrease to the normal level at T35 and later time points (Fig. 1b). Similar short-term blooms of particular microbes also occur in samples from other individuals (Supplementary Fig. 2).

### Dependence of gut microbiota composition on the time interval between sampling points

**Fig. 2:**
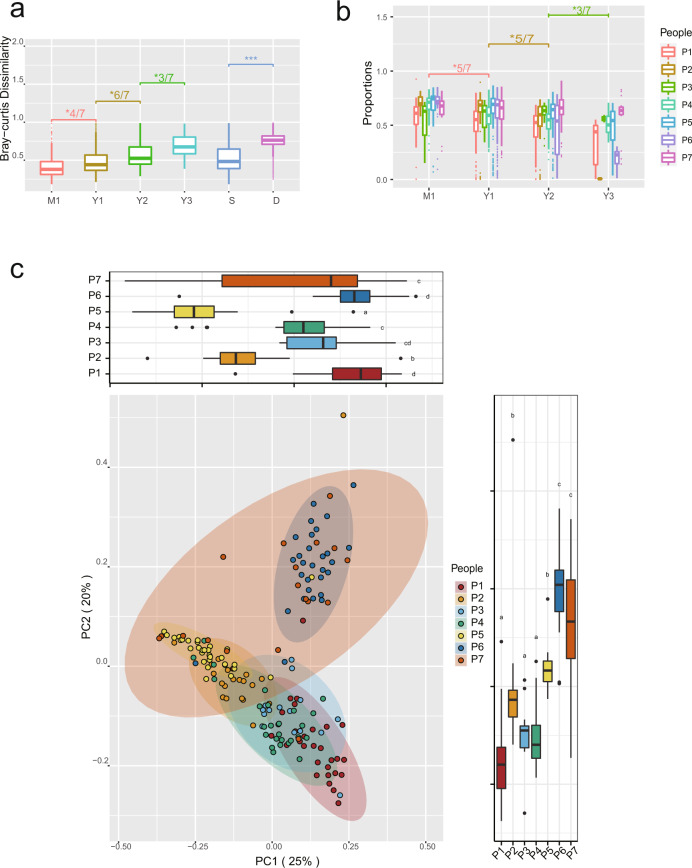
Sample comparsions for gut microbiota composition. **a** Boxplots showing the Bray–Curtis dissimilarities between samples from four groups (M1/Y1/Y2/Y3). M1 denotes comparisons from the same subject collected within a short time interval (1 month); Y1 and Y2 denote comparisons from the same subject collected within intermediate time intervals (Y1, 2–12 months; Y2, 13–24 months); and Y3 denotes comparisons from the same subject collected within a longer time interval (>24 months). S denotes comparisons from samples obtained within the same participant; D denotes comparisons between samples from different participants. P participant. ***p < 0.001; * 4/7: The significance (*p* < 0.05) applies to 4 of 7 individuals; *6/7: The significance (*p* < 0.05) appies to 6 of 7 individuals; *3/7: The significance (*p* < 0.05) applies to 3 of 7 individuals. **b** Boxplots showing the proportions of source bacteria retained in a given microorganism community in the fecal samples. *5/7: The significance (*p* < 0.05) applies to 5 of 7 individuals; *3/7: The significance (*p* < 0.05) applies to 3 of 7 individuals. **c** PCoA diagram of samples from seven participants at the genus level. We uses the letters **a**, **b** and **c** to show statistically significant differences between groups. For all groups with the same letter, the difference between the means is not statistically significant. If two variables have different letters, they are significantly different. Boxplots display the distribution of data based on the five number summary: minimum, first quartile, median, third quartile, and maximum.

Even though the gut microbiota of the subjects fluctuates over time, samples from the same individual were less dissimilar than those from different individuals. We calculated the Bray–Curtis dissimilarities between the samples collected for each subject. We found that Bray–Curtis dissimilarities between samples originating from the same individual were significantly lower than those between samples originating from different individuals (Fig. 2a). Significant differences between groups were both determined using the Wilcoxon test and Permutation test, and cases with *p* < 0.05 were considered statistically significant. Principal Co-ordinates Analysis (PCoA) based on Bray–Curtis dissimilarities for bacterial community at the genus level also supported that samples from the same individual were clustered together (Fig. 2c).

We calculated the Bray–Curtis dissimilarities between pairs of samples collected at different time points for each subject (Supplementary Fig. 3) and arranged the pairs of samples from the same individual into four groups, labelled, M1, Y1, Y2, and Y3. Specifically, M1 consisted of pairs of samples that were collected within two consecutive months, while Y1 and Y2 contained intermediate time intervals, involving samples that were collected within 2–12 and 13–24 months, respectively. Finally, Y3 contained pairs of samples collected with a time interval of over 24 months. For the 171 fecal samples from the seven individuals recruited in this study, we compared all these samples in pairs, and get 262 comparisons collected within two consecutive months (M1 group), 2132 comparisons for two samples collected within 2–12 months (Y1 group), 1442 comparisons for two samples collected within 13–24 months (Y2 group), and 414 comparisons for two samples with a time interval of over 24 months (Y3 group).

Based on the calculated Bray-Curtis dissimilarities between the samples and the comparison between groups, differences in gut microbiota between samples increased over time at the genus, OTU and ASV levels (Supplementary Fig. 3). We performed separate significance tests for each participant, to determine whether there was a significant increase in variation over time for that individual (Fig. 2a; Supplementary Table 2). For each individual, we permuted the time points, and calculated the Wilcoxon test statistic for the Bray-Curtis dissimilarities on these permuted data. We repeated this 1000 times and found critical values by selecting appropriate percentages from this distribution. We compared the Wilcoxon statistic for the observed Bray-Curtis dissimilarities to these critical values. A significant increase in Bray-Curtis dissimilarities between M1 and Y3 groups was observed in all individuals, with the exception of P3.

### Persistence of gut bacteria over time

SourceTracker2^17^, which uses a Bayesian approach to estimate the shared proportion of source organisms in a given community, has been successfully applied to accurately quantify multiple sources with low relative abundances in sink communities^17^ and for the identification of fecal pollution in recreational freshwater^18^ as well as coastal water^19^. In this study, it was applied to estimate the shared proportion of bacteria remaining in the samples across two time points. We observed an average shared proportion value of 63.5% for samples collected from the same individual in the preceding month, and as the time interval between the time points increased, this shared proportion value decreased (Fig. 2b). For bacteria, it was 58.5, 51.8, and 40.7% for samples collected within a year, 2 years, and over the long-term (Y3: > 24 months), respectively (Fig. 2b). Therefore, over time, the shared proportion of bacteria from the initial samples that were retained in the gut decreased over time.

### Differences in gut microbiota bacterial composition

**Fig. 3:**
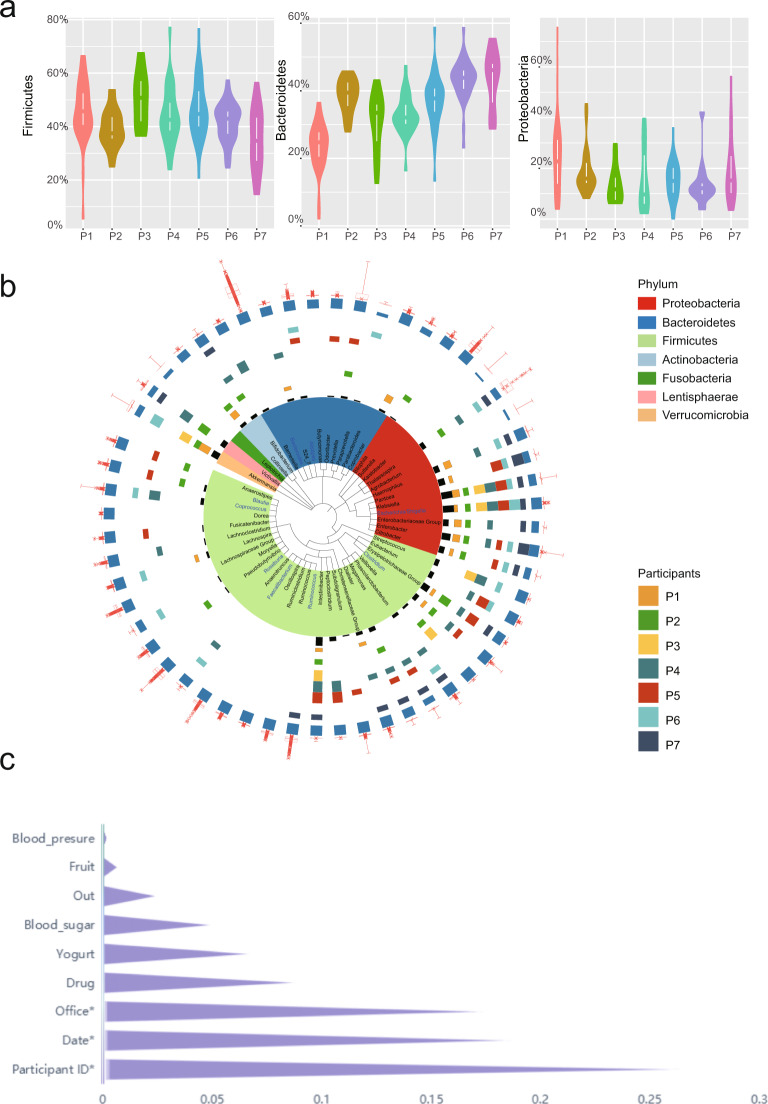
Differences in gut microbiota bacterial composition. **a** Violin plots presenting the abundances of Firmicutes, Bacteroidetes, and Proteobacteria in fecal samples obtained from the seven participants. P, participant. **b** Taxonomy tree of 58 genera identified in this study. Three bar charts from inside to outside show the total number of short-term blooms (Black bar chart), the number of short-term blooms in 7 participants (P1-P7) and the percentage of samples in which genus was detected in this study (Blue bar chart). The outermost red box plot shows the relative abundance of each genus in all samples. Boxplots display the distribution of data based on the five number summary: minimum, first quartile, median, third quartile, and maximum. General labeled in blue text are core genera identified in all samples. The background color represents the phylum. **c** Scores corresponding to the influence of nine factors (participant ID, date, workspace/office, drug use, yogurt consumption, fruit consumption, travelling abroad history, blood pressure, and blood sugar) on gut microbiota composition over time.

We identified 8 phyla and 58 genera in the 171 collected fecal samples. Of these, only 3 phyla (Firmicutes, Bacteroidetes, and Proteobacteria) and 9 genera (*Bacteroides*, *Roseburia*, *Ruminococcus*, *Faecalibacterium*, *Pseudobutyrivibrio*, *Blautia*, *Lachnoclostridium*, *Oscillospira*, and *Lachnospira*) were present in all the samples; the remainder were present in only a fraction of samples. For example, the genus *Klebsiella* was identified in all P3 samples, but only in 55.6% of P4 samples. These findings provide further evidence of the instability of the gut microbiota composition over time, as well as the differring degrees of fluctuation for the abundance of difference bacteria, even for core phyla (Fig. 3a).

At the genus level, we used the number of short-term blooms to show the fluctuation of the relative abundances of the bacteria for each participant (Fig. 3b). Of the 58 genera identified in this study, the percentage of 45 genera (77.6%) fluctuated via short-term blooms. We counted the number of short-term blooms for each genus and each participant (Fig. 3b) and found that fluctuation of the relative abundances of the bacteria for each participant is not identical. For example, *Clostridium* was identified as a core genus in our previous study, which involved healthy Chinese individuals^10^ and individuals from other countries^20^, and in this study, was detected in all samples, except for one. The short-term blooms of *Clostridium* were identified in 4 individuals at different timepoints (P1, P2, P4 and P5, Fig. 1b and Supplementary Fig. 2) but not in P3, P6 and P7, indicating that *Clostridium* undergoes fluctuation to varying degrees in the intestinal tract of different participants (Fig. 3b).

### The effect of external factors on the gut microbiota

A mantel test was used to assess the association between the gut microbiome of the study participants and nine predictor variables for each participant, collected via our survey form (participant ID, date, workspace/office,drug use, yogurt consumption, fruit consumption, travel history, blood pressure, and blood sugar). This test was performed using the R packages (corrplot^21^ and mantel^22^). Our investigation identified the individual (sample host) as the most significant influencing factor, followed by time (sample timepoint). As shown in Fig. 3c, yogurt and fruit consumption as well as blood pressure and blood sugar level did not significantly co-vary with the human gut microbiota.

## Discussion

This study, specifically geared towards Chinese people, focused on determining the characteristics of gut microbiota in healthy Chinese people across multiple years, and we found that the healthy gut microbiota of different individuals is not consistent, which was also supported by other previous studies^10^. By analyzing 171 fecal samples collected from 7 volunteers and identifying the microbiota to phylum/genus/species/OTU/ASV level, we observed that the bacterial composition from each subject was variable at each of these taxonomic levels (Fig. 1 and Supplementary Fig. 1). PCoA analysis for gut microbiota also supported that samples from the same individual were clustered together, but separately from samples from the other individuals (Fig. 2c). Further, by comparing the effects of several factors (individual, time, workspace/office, drug use, yogurt consumption, fruit consumption, travel history, blood pressure, and blood sugar), on the gut microbiota composition, we observed that individualistic differences were primarily responsible for the largest proportion of changes in gut microbiota composition.

Healthy is not equivalent to stable. Our observational study revealed that the composition of human gut microbiota changes over time. Firstly, we observed that an individual’s microbiota can fluctuate on different levels (Phylum/Genus/Species/OTU/ASV). At the genus level, there were 83 samples (48.5%) with short-term blooms of particular bacterial genera. Secondly, changes in gut microbiota composition increased over time. The difference between samples collected within a short time interval was significantly lower than that between samples collected within longer intervals (Fig. 2a). On average, the source homogeneity corresponding to the M1 group was 63.5%; as the time interval increased, this value decreased to 40.7% (Y3: >24 months).

For samples from P6 and P7 after timepoint 28, we observed a shift in microbial composition, just as shown in Fig. 1 and Supplementary Fig. 1. After excluding all samples after timepoint 28 from 7 participants, the Bray-Curtis dissimilarities corresponding to the short-time group (M1) were still significantly lower than those corresponding to the long-time groups (Y1 and Y2), just as shown in Supplementary Fig. 4.

The fluctuation of healthy gut microbiota could be caused by the transiently positive bacterial strains. In this study, we identified a total of 8 phyla and 58 genera in the 171 collected fecal samples. However, only 3 phyla (Firmicutes, Bacteroidetes, and Proteobacteria) and 9 genera (*Bacteroides*, *Roseburia*, *Ruminococcus*, *Faecalibacterium*, *Pseudobutyrivibrio*, *Blautia*, *Lachnoclostridium*, *Oscillospira*, and *Lachnospira*) were present in all the samples; the remainder were missing in different proportions, which supported the hypothesis that the healthy gut microbiota contains a certain proportion of transient bacterial strains. These transient bacterial strains could come from the outside, such as food or human external environment, but this would need to be further proven in a later study.

Even for core bacterial phylum and genera, the fluctuation still existed (Fig. 3a), but they fluctuated to varying degrees. *Bacteroides*, which metabolizes polysaccharides and oligosaccharides, providing nutrition and vitamins to the host and other intestinal microbial residents^23^, was detected in all the collected fecal samples and showed the differring degrees of fluctuation for the abundance in each participant (Fig. 3a, b).

Limitations of the study included a small sample size and the use of only 16 S rRNA gene sequencing. A limitation of 16 S rRNA gene sequencing is that it provides information on the relative abundance of bacterial taxa in samples but not absolute quantitative abundance^24^. In our future studies, we will process a larger number of samples, adding whole-genome sequencing data, as well as developing new analysis methods to get absolute quantitative abundance of particular microbes in the gut microbiome. These notwithstanding, our results indicated that gut microbiota characteristics change over time.

In summary, our findings revealed the occurrence of changes in gut microbiota characteristics over time and suggested that the influence of time should be considered when gut microbiota is used as a tool for the evaluation of an individual’s health and for the diagnosis of diseases.

## Methods

### Sample collection

Our study involved fecal samples obtained from healthy Chinese individuals, who provided written informed consent prior to enrollment in this study. Each month between October 2016 and May 2020, we collected fecal samples from healthy Chinese individuals residing in Beijing, China. The eligibility criteria for participation in this study were as follows: (1) Aged 18 years and above, but not >70 years old at the time of enrollment; (2) Body mass index in the range 16–30; (3) Healthy and willing as well as able to provide stool specimens and fill a detailed questionnaire each month; (4) Blood pressure <140/90 mmHg and blood sugar level after meal <11.1 mmol/L; (5) Participants without any history of cancer, tuberculosis, illnesses requiring surgery, or 40 other conditions. Details regarding the characteristics of the subjects are provided in the Supplementary Table 1. To ensure the follow-up data analysis can be carried out over a multi-year time period, samples from individuals who participated in the sampling <15 times were filtered out.

At each sampling time, the volunteers were asked to complete a detailed questionnaire with information on their age, occupation, and drug/medical history. The same information was also collected for their immediate families. Time points with a small number of suitable samples (<3) were also excluded. Finally, 171 suitable fecal samples from seven individuals were collected. The number of samples collected from each subject, corresponding to a total of 37 time points, varied in the range 15–31 (Mean, 24.7; Median, 27).

The daily habits (smoking, physical exercise, and fruit and alcoholic beverage consumption) of the subjects were also evaluated each month to determine their health status. Additionally, their heights, weights, blood pressure values, and blood glucose levels were recorded onsite every month. Thereafter, we constructed a detailed report on candidate-specific factors that could affect their gut microbiome. Detailed information in this regard is provided in Supplementary Table 1.

The fecal samples were collected using a disposable bedpan provided to the volunteers rather than obtaining samples from a flush toilet; this was to avoid contamination by toilet water. Further, these samples were collected within 24 h after completing the tests and questionnaire for each sampling time point.

### Sample preparation and sequencing analysis

DNA was extracted from the fecal samples within 24 h after collection using the QIAamp Fast DNA Stool Mini Kit (Qiagen, Hilden, Germany) according to the manufacturer’s instructions. The V3–V4 region of the 16 S rRNA in each sample was amplified using the primers 341 F 5′-CCTACGGGNGGCWGCAG-3′ and 805 R 5′-GACTACHVGGGTATCTAATCC-3′. Thereafter, the amplified fragments were sequenced on an Illumina MiSeq platform (San Diego, CA, USA) with paired-end sequencing. Thus, reads consisting of 300 base pairs were generated.

### Data analysis

The low-quality (<Q20) bases at the end of the reads were trimmed off, and only reads with ≥200 base pairs were retained as high-quality reads. The number of high-quality reads for each sample was above 30,000, and the percentage of bases with quality values higher than Q30 was above 80%.

For qualified samples, the number of operational taxonomic units (OTUs) and the relative abundance of each bacterial Phylum/Genus/Species corresponding to the qualified samples were calculated using parallel-meta3 software^25^, which is based on the GreenGenes-13-8 database and the default parameters (97% identity level). The number of amplicon sequence variants (ASVs) were calculated using dada2^26^, with the default parameters.

If the abundance of a particular genus in one sample is >5 times the average abundance of the genus in samples from the same individual, then that sample is considered to be a short-term bloom for that genus.

The variability of bacteria relative abundance for the determination of differences between samples with respect to community composition was determined by calculating the Bray-Curtis dissimilarities between pairs of samples from a given individual, with sequences clustered at each of the genus, OTU and ASV levels, using the R package Vegan^22^ (The R Project for Statistical Computing, Vienna, Austria). We also used the Vegan package for PCoA analysis in this study and used ggplot2^27^ for drawing figures.

Because the pairs of samples are not independent, the usual critical values of the Wilcoxon test are not valid. We therefore use a permutation test to calculate the significance values. For each individual, we permute the time points, and calculate the Wilcoxon test statistic for the Bray-Curtis dissimilarities on this permuted data. We repeat this 1000 times and find critical values by selecting appropriate percentiles from this distribution. We also perform separate significance tests for each participant, to determine whether increased variation over time was a common feature of all individuals, or whether only some individuals experienced it. The script used in this study for statistical calculation, such as the Wilcoxon test and Permutation test, were released in the Github (https://github.com/zhangwencdc/Guthealthy16S/).

Next, we used the SourceTracker2 software^17^ to estimate the proportion of microbiota retained over time via comparison with the proportions corresponding to the samples collected at earlier time points. The data obtained were presented using R packages, ggplot2^27^ and ggsignif^28^. We perform the same significance test based on Permuation and Wilcoxon test as above.

We used iTOL^29^, an online tool for display, manipulation, and annotation of taxonomic trees, to integrate and visualize taxonomy information, and abundance changes of the 58 genera.

### Reporting summary

Further information on research design is available in the Nature Portfolio Reporting Summary linked to this article.

## Supplementary information

Supplemental Material

Description of Additional Supplementary Files

Supplemental Data 1

Reporting Summary

## Data Availability

All sequencing data have been deposited in the NCBI SRA database (SRR14066491 to SRR14066400) and the public database of pathogenic microorganisms (http://data.mypathogen.org/index). The source data used for graphs and charts has been released in Figshare (10.6084/m9.figshare.21556704.v2).

## Code availability

The source code used in this study were released in GitHub (https://github.com/zhangwencdc/Guthealthy16S/).The licence of the codes in under GPL v3 or later (http://www.gnu.org/licenses/gpl-3.0.html).
