# Supplementary material for: Time-scale analysis of the long-term variability of human gut microbiota characteristics in Chinese individuals
Source: Commun Biol. 2022 Dec 23;5:1414. doi: 10.1038/s42003-022-04359-9 (PMC9789056; doi:10.1038/s42003-022-04359-9)
Supplement: Supplementary file 1 — Supplemental Material [file 42003_2022_4359_MOESM1_ESM.pdf]

**Supplementary Table 1. Personal Information Questionnaire**

## Personal Information Questionnaire

- ID:
- **Basic information**

| | | | | | |
|---|---|---|---|---|---|
| Name | | Gender | | | |
| Age | | Height(cm) | | Weight (kg) | |
| Blood pressure | | | | | |
| Blood sugar after diet | | | | | |

- **History of drug use or disease in the past month**

| | Other |
|---|---|
| Have you taken antibiotics in the past month (such as cephalosporins, penicillin, etc.)?<br>Yes☐ No☐ | |
| Have you used infusion therapy in the past month?<br>Yes☐ No☐ | |
| Have you had pain, hemorrhoids, or blood in the stool in the past month?<br>Yes☐ No☐ | |
| Have you had a rectal examination or treatment in the past month?<br>Yes☐ No☐ | |
| Have you had a case of watery or egg like diarrhea in the past month?<br>Yes☐ No☐ | |
| Has there been a period of more than 3 days in the past month when you did not defecate?<br>Yes☐ No☐ | |
| Have you taken any drugs to help defecate in the past month?<br>Yes☐ No☐ | |
| Have you taken any drugs to treat diarrhea in the past month?<br>Yes☐ No☐ | |
| Have you had surgery in the past month?<br>Yes☐ No☐ | |
| Have you taken any other drugs in the past month?<br>Yes☐ No☐ | |

- **Diet and lifestyle habit survey**

| | | | | |
|---|---|---|---|---|
| How often have you eaten lactic acid products (such as yogurt) in the | ☐ Every day | ☐ Three or more times | ☐ One or two times | ☐Never |

| | | | | |
|---|---|---|---|---|
| past month? | | | | |
| How often have you eaten fruit in the past month? | ☐ Every day | ☐Once per two or three days | ☐Occasionally | ☐Never |
| How often have you smoked? | ☐More than 2 cigarettes a day | ☐Once per two or three days | ☐Occasionally | ☐Never |
| How often have you drunk alcohol? | ☐ Every day | ☐Once per two or three days | ☐Occasionally | ☐Never |
| How often have you exercised in the past month | ☐ More than 1 hour per day | ☐one or two times per week | ☐Occasionally | ☐Never |
| How many hours did you sleep in the past month | ☐more than 8 hours per one day | ☐6-8 hours per day | ☐4-6 hours per day | ☐<4 hours per day |
| What do you mainly eat? | ☐light ☐salty | ☐sweet | ☐hot and spicy | ☐fried food |

- **Working and living environment**

- ☐Noise
- ☐Electromagnetic radiation
- ☐Dust pollution
- ☐Chemical pollution
- ☐Air pollution
- ☐Pollution of building decoration
- ☐Cooking fume pollution
- ☐Biochemical Reagent
- ☐Other pollution
- ☐None of the above

- **Medical History**

| | | | | |
|---|---|---|---|---|
| ☐Hypertension | ☐Diabetes | ☐Cirrhosis | ☐History of obesity | ☐Helicobacter pylori infection |
| ☐Coronary heart disease | ☐Hyperthyroidism | ☐Pancreatic disease | ☐Peripheral vascular disease | ☐Chronic cholecystitis |
| ☐Rheumatic heart disease | ☐Anemia | ☐Acute and chronic nephritis | ☐Heart failure | ☐Chronic breast disease |
| ☐Congenital heart disease | ☐Epilepsy | ☐Connective tissue disease | ☐Chronic obstructive pulmonary disease | ☐Blood lipid abnormality |

| | | | | |
|---|---|---|---|---|
| ☐Cardiomyopathy | ☐Mental Disease | ☐Sexually transmitted disease | ☐Osteoporosis | ☐Elevated uric acid |
| ☐Bronchiectasis | ☐Neurosis | ☐Cancer | ☐Gout | ☐History of severe allergies |
| ☐Bronchial asthma | ☐History of drug abuse | ☐History of surgery | ☐Rheumatoid arthritis | ☐Hereditary disease |
| ☐Emphysema | ☐Acute and chronic hepatitis | ☐History of severe trauma | ☐Cerebral apoplexy | ☐Other disease |
| ☐Peptic ulcer | ☐Tuberculosis | ☐HPV | ☐Asthma | |

- **Medical history of immediate family**

| | | | | |
|---|---|---|---|---|
| ☐Hypertension | ☐Diabetes | ☐Cirrhosis | ☐History of obesity | ☐Helicobacter pylori infection |
| ☐Coronary heart disease | ☐Hyperthyroidism | ☐Pancreatic disease | ☐Peripheral vascular disease | ☐Chronic cholecystitis |
| ☐Rheumatic heart disease | ☐Anemia | ☐Acute and chronic nephritis | ☐Heart failure | ☐Chronic breast disease |
| ☐Congenital heart disease | ☐Epilepsy | ☐Connective tissue disease | ☐Chronic obstructive pulmonary disease | ☐Blood lipid abnormality |
| ☐Cardiomyopathy | ☐Mental Disease | ☐Sexually transmitted disease | ☐Osteoporosis | ☐Elevated uric acid |
| ☐Bronchiectasis | ☐Neurosis | ☐Cancer | ☐Gout | ☐History of severe allergies |
| ☐Bronchial asthma | ☐History of drug abuse | ☐History of surgery | ☐Rheumatoid arthritis | ☐Hereditary disease |
| ☐Emphysema | ☐Acute and chronic hepatitis | ☐History of severe trauma | ☐Cerebral apoplexy | ☐Other disease |
| ☐Peptic ulcer | ☐Tuberculosis | ☐HPV | ☐Asthma | |

**Supplementary Table 2.** The Permutation test for group difference (M1, Y1, Y2 and Y3). Significance (* p<0.05; ** p<0.01)

| People | Group Compare | Genus | OTU | ASV |
|---|---|---|---|---|
| P1 | M1 vs Y1 | | | |
| P1 | Y1 vs Y2 | * | ** | ** |
| P1 | Y2 vs Y3 | | | |
| P1 | M1 vs Y3 | | * | * |
| P2 | M1 vs Y1 | | ** | * |
| P2 | Y1 vs Y2 | ** | ** | ** |
| P2 | Y2 vs Y3 | | | |
| P2 | M1 vs Y3 | ** | ** | ** |
| P3 | M1 vs Y1 | | | |
| P3 | Y1 vs Y2 | | | |
| P3 | Y2 vs Y3 | | | |
| P3 | M1 vs Y3 | | | |
| P4 | M1 vs Y1 | ** | ** | ** |
| P4 | Y1 vs Y2 | * | ** | ** |
| P4 | Y2 vs Y3 | | * | * |
| P4 | M1 vs Y3 | ** | ** | ** |
| P5 | M1 vs Y1 | | | |
| P5 | Y1 vs Y2 | ** | ** | ** |
| P5 | Y2 vs Y3 | | | |
| P5 | M1 vs Y3 | * | * | * |
| P6 | M1 vs Y1 | ** | ** | ** |
| P6 | Y1 vs Y2 | | ** | ** |
| P6 | Y2 vs Y3 | | ** | ** |
| P6 | M1 vs Y3 | * | ** | ** |
| P7 | M1 vs Y1 | | * | |
| P7 | Y1 vs Y2 | | * | ** |
| P7 | Y2 vs Y3 | ** | ** | ** |
| P7 | M1 vs Y3 | ** | ** | ** |

**Supplementary Figure 1.** (**a**) Percentage of different bacterial phylum in fecal samples from seven participants at different time points between 2016 and 2020. (b) Percentage of different bacterial species in fecal samples from seven participants. (c) Percentage of different OTUs in fecal samples from seven participants. (d) Percentage of different ASVs in fecal samples from seven participants. Only taxa with abundance >=0.1% were shown. T, time point. The detailed information for Time points was in Supplementary Material 1. * represented the short time bloom of particular microbes at this time point on phylum and species level.

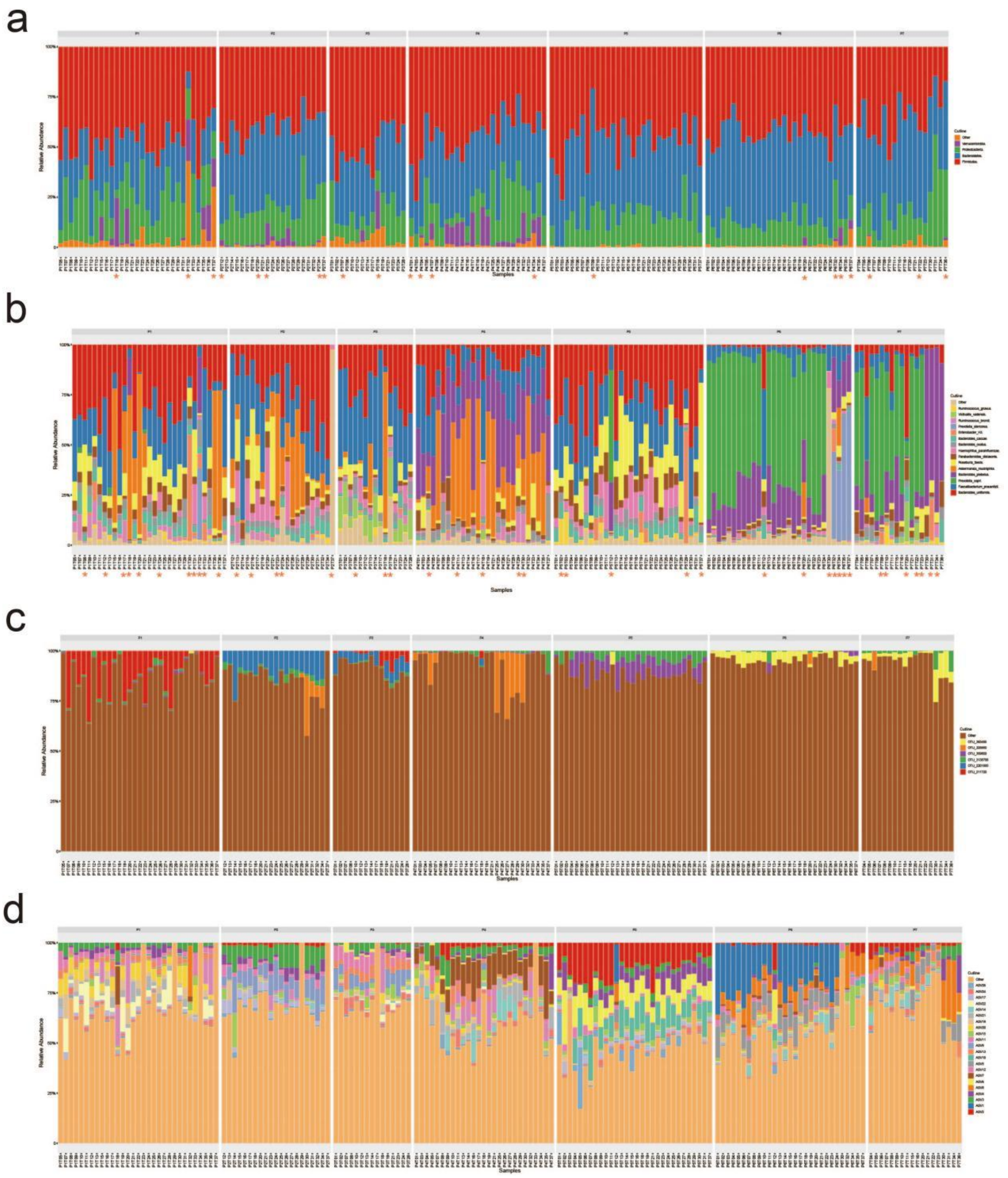

**Supplementary Figure 2.** The line charts for the short term blooms of particular microbes in samples from individual (P2-P7).

P2

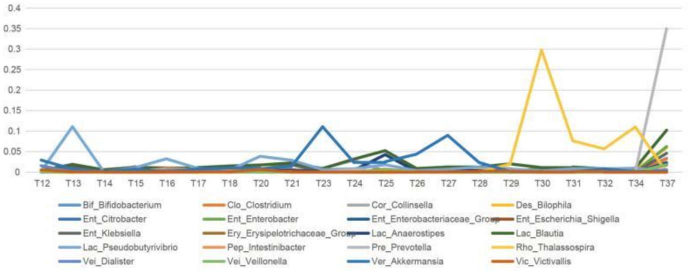


P3

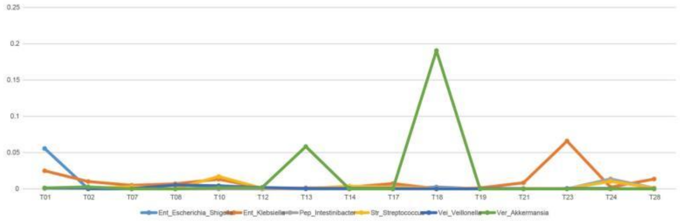

P4

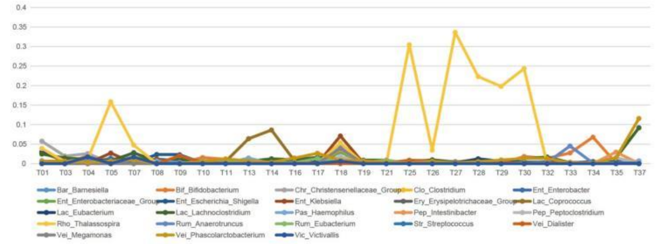


P5

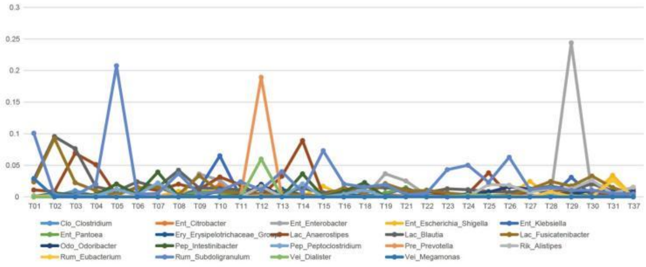


P6

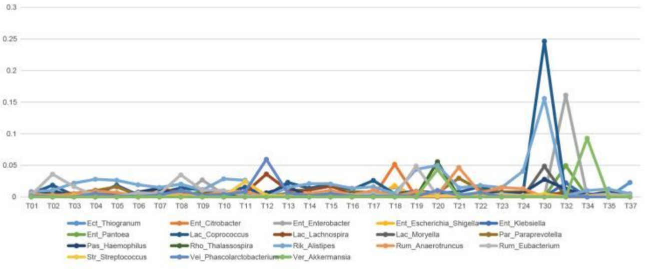


P7

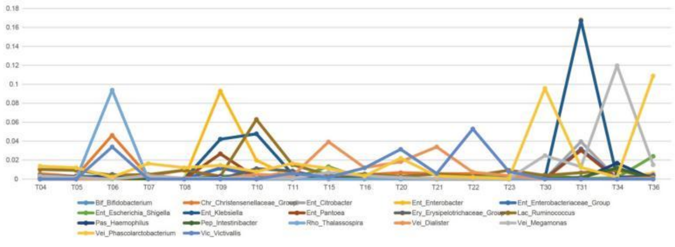

**Supplementary Figure 3.** (**a**) Boxplots showing the Bray–Curtis dissimilarity on genus level for samples from seven participants (P1-P7). (**b**) Boxplots showing the Bray–Curtis dissimilarity on OTU level for samples from seven participants (P1-P7). (**c**) Boxplots showing the Bray–Curtis dissimilarity on ASV level for samples from seven participants (P1-P7). Boxplots show minimum, first quartile, median, third quartile and maximum value of the dataset.

a

b

c

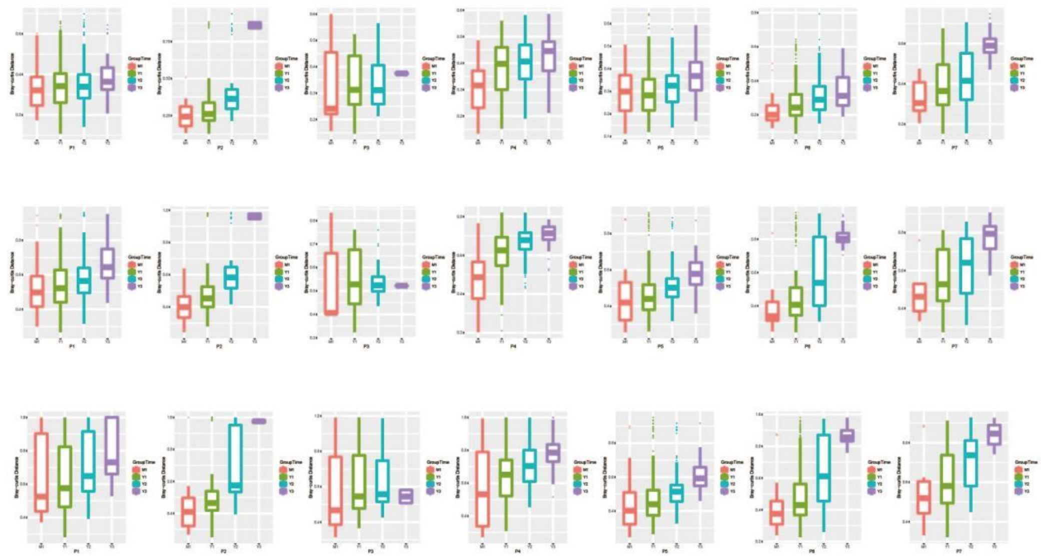

**Supplementary Figure 4.** (**a**) Boxplots showing the Bray–Curtis dissimilarity on genus level for samples (before T28) from seven participants (P1-P7) (**b**) Boxplots showing the Bray–Curtis dissimilarity on OTU level for samples (before T28) from seven participants (P1-P7). (**c**) Boxplots showing the Bray–Curtis dissimilarity on ASV level for samples (before T28) from seven participants (P1-P7). (**d**) Boxplots showing the Bray–Curtis dissimilarity between samples (before T28) from 4 groups (M1/Y1/Y2/Y3) on genus level. (**e**) Boxplots showing the Bray–Curtis dissimilarity between samples (before T28) from 4 groups (M1/Y1/Y2/Y3) on OTU level. (**f**) Boxplots showing the Bray–Curtis dissimilarity between samples (before T28) from 4 groups (M1/Y1/Y2/Y3) on ASV level. Boxplots show minimum, first quartile, median, third quartile and maximum value of the dataset.

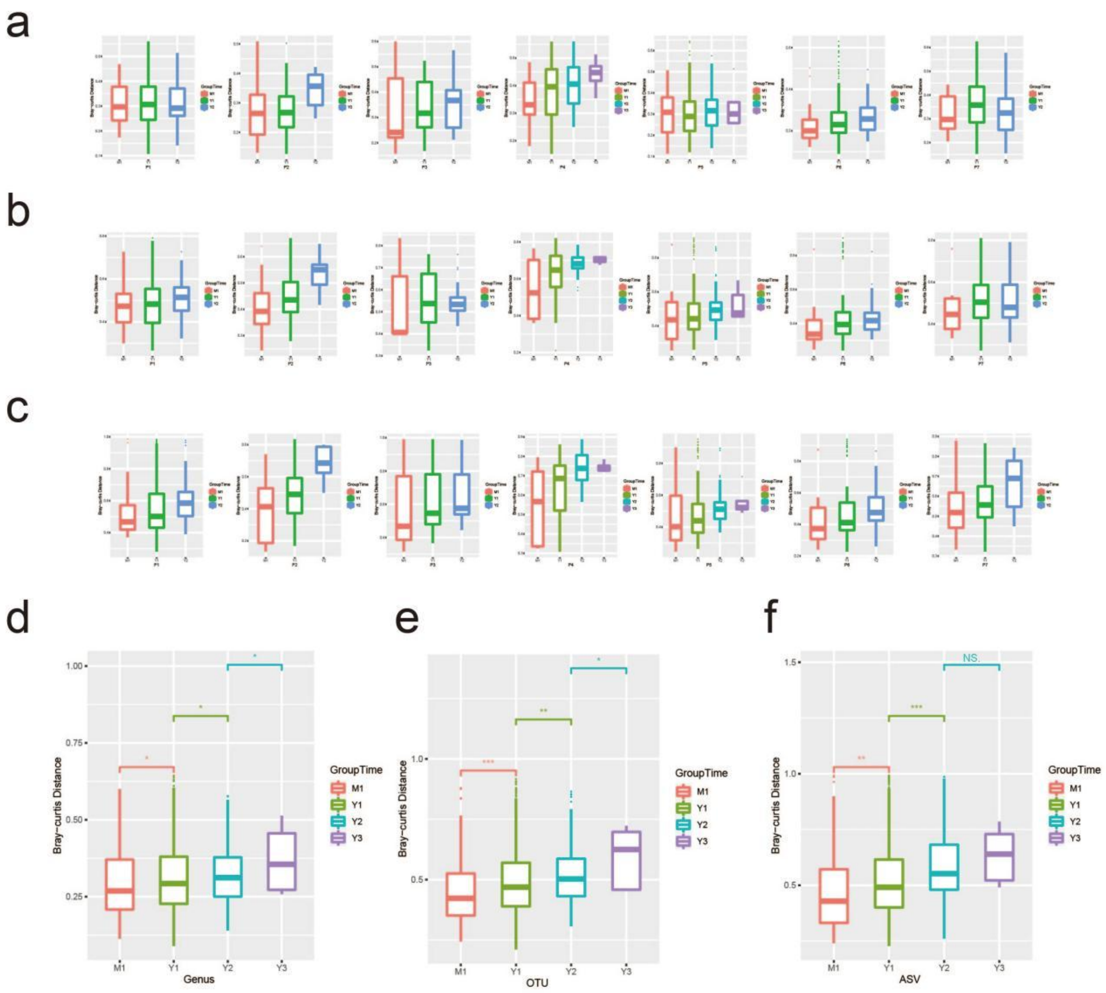

a
b
c
d
e
f
Bray−curtis Distance
GroupTime
M1
Y1
Y2
Y3
Genus
OTU
ASV
1.00
0.75
0.50
0.25
1.0
0.5
1.5
NS.
