# Supplementary material for: Time-scale analysis of the long-term variability of human gut microbiota characteristics in Chinese individuals
Source: Commun Biol. 2022 Dec 23;5:1414. doi: 10.1038/s42003-022-04359-9 (PMC9789056; doi:10.1038/s42003-022-04359-9)
Supplement: Supplementary file 2 — Description of Additional Supplementary Files [file 42003_2022_4359_MOESM2_ESM.pdf]

Description of Additional Supplementary Files

File name: Supplementary Data 1
Description: Detailed sample information.
