# Supplementary material for: Time-scale analysis of the long-term variability of human gut microbiota characteristics in Chinese individuals
Source: Commun Biol. 2022 Dec 23;5:1414. doi: 10.1038/s42003-022-04359-9 (PMC9789056; doi:10.1038/s42003-022-04359-9)
Supplement: Supplementary file 4 — Reporting Summary [file 42003_2022_4359_MOESM4_ESM.pdf]

nature portfolio

Corresponding author(s): *Double-blind peer review submissions: write DBPR and your manuscript number here instead of author names.*

Last updated by author(s): *YYYY-MM-DD*

Reporting Summary

Nature Portfolio wishes to improve the reproducibility of the work that we publish. This form provides structure for consistency and transparency in reporting. For further information on Nature Portfolio policies, see our Editorial Policies and the Editorial Policy Checklist.

Statistics

For all statistical analyses, confirm that the following items are present in the figure legend, table legend, main text, or Methods section.

n/a	Confirmed	
☐	☒	The exact sample size (*n*) for each experimental group/condition, given as a discrete number and unit of measurement
☐	☒	A statement on whether measurements were taken from distinct samples or whether the same sample was measured repeatedly
☐	☒	The statistical test(s) used AND whether they are one- or two-sided<br>*Only common tests should be described solely by name; describe more complex techniques in the Methods section.*
☒	☐	A description of all covariates tested
☐	☒	A description of any assumptions or corrections, such as tests of normality and adjustment for multiple comparisons
☐	☒	A full description of the statistical parameters including central tendency (e.g. means) or other basic estimates (e.g. regression coefficient) AND variation (e.g. standard deviation) or associated estimates of uncertainty (e.g. confidence intervals)
☒	☐	For null hypothesis testing, the test statistic (e.g. *F*, *t*, *r*) with confidence intervals, effect sizes, degrees of freedom and *P* value noted<br>*Give P values as exact values whenever suitable.*
☒	☐	For Bayesian analysis, information on the choice of priors and Markov chain Monte Carlo settings
☒	☐	For hierarchical and complex designs, identification of the appropriate level for tests and full reporting of outcomes
☒	☐	Estimates of effect sizes (e.g. Cohen's *d*, Pearson's *r*), indicating how they were calculated

Our web collection on statistics for biologists contains articles on many of the points above.

Software and code

Policy information about availability of computer code

Data collection	All sequencing data have been deposited in the NCBI SRA database (SRR14066491 to SRR14066400) and the public database of pathogenic microorganisms (http://data.mypathogen.org/index).
Data analysis	The script used in this study were released in the Github (https://github.com/zhangwencdc/Guthealthy16S/). We used SourceTracker2 software 17 to estimate the proportion of microbiota retained over time via comparison with the proportions corresponding to the samples collected at a preceding time point. The data obtained were presented using R packages, ggplot226 and ggsignif27. We perform the same significance test based on Permuation and Wilcoxon test as above.<br>We used iTOL, an online tool for display, manipulation, and annotation for taxonomy tree

For manuscripts utilizing custom algorithms or software that are central to the research but not yet described in published literature, software must be made available to editors and reviewers. We strongly encourage code deposition in a community repository (e.g. GitHub). See the Nature Portfolio guidelines for submitting code & software for further information.

Data

Policy information about availability of data

All manuscripts must include a data availability statement. This statement should provide the following information, where applicable:

- Accession codes, unique identifiers, or web links for publicly available datasets
- A description of any restrictions on data availability
- For clinical datasets or third party data, please ensure that the statement adheres to our policy

All sequencing data have been deposited in the NCBI SRA database (SRR14066491 to SRR14066400) and the public database of pathogenic microorganisms (http://data.mypathogen.org/index).

Human research participants

Policy information about studies involving human research participants and Sex and Gender in Research.

Reporting on sex and gender	*Use the terms sex (biological attribute) and gender (shaped by social and cultural circumstances) carefully in order to avoid confusing both terms. Indicate if findings apply to only one sex or gender; describe whether sex and gender were considered in study design whether sex and/or gender was determined based on self-reporting or assigned and methods used. Provide in the source data disaggregated sex and gender data where this information has been collected, and consent has been obtained for sharing of individual-level data; provide overall numbers in this Reporting Summary. Please state if this information has not been collected. Report sex- and gender-based analyses where performed, justify reasons for lack of sex- and gender-based analysis.*
Population characteristics	*Describe the covariate-relevant population characteristics of the human research participants (e.g. age, genotypic information, past and current diagnosis and treatment categories). If you filled out the behavioural & social sciences study design questions and have nothing to add here, write "See above."*
Recruitment	*Describe how participants were recruited. Outline any potential self-selection bias or other biases that may be present and how these are likely to impact results.*
Ethics oversight	*Identify the organization(s) that approved the study protocol.*

Note that full information on the approval of the study protocol must also be provided in the manuscript.

Field-specific reporting

Please select the one below that is the best fit for your research. If you are not sure, read the appropriate sections before making your selection.

☒ Life sciences ☐ Behavioural & social sciences ☐ Ecological, evolutionary & environmental sciences

For a reference copy of the document with all sections, see nature.com/documents/nr-reporting-summary-flat.pdf

Life sciences study design

All studies must disclose on these points even when the disclosure is negative.

Sample size	171 fecal samples from seven individuals were collected in this study.
Data exclusions	The eligibility criteria for participation in this study were as follows: (1) Aged 18 years and above, but not more than 70 years old at the time of enrollment; (2) Body mass index in the range 16–30; (3) Healthy and willing as well as able to provide stool specimens and fill a detailed questionnaire each month; (4) Blood pressure < 140/90 mmHg and blood sugar level after meal < 11.1 mmol/L; (5) Participants and immediate family without any history of cancer, tuberculosis, surgery, or 40 other kinds of diseases. Details regarding the characteristics of the subjects are provided in the Supplemental Material 2. To ensure the follow-up data analysis can be carried out on years level, samples from individuals who participated in the sampling for fewer than 15 times were filtered out.<br>At each sampling time, the volunteers were asked to complete a detailed questionnaire with information on their age, occupation, and drug/medical history. The same information was also collected for their immediate families. When the participants provided information regarding a history of infusion therapy in the past month or constipation, hemorrhoids, and blood in the stool, or a history of rectal exams/watery or egg flower, or cold in the past month, the samples collected were filtered out. If they used any antibiotics within the last month, the samples collected were also excluded. Time points with a small number of suitable samples (<3) were also excluded.
Replication	Each month between October 2016 and May 2020, we collected fecal samples.
Randomization	Each month between October 2016 and May 2020, we collected fecal samples.
Blinding	The investigators were blinded to group allocation during sample and sequencing.

Reporting for specific materials, systems and methods

We require information from authors about some types of materials, experimental systems and methods used in many studies. Here, indicate whether each material, system or method listed is relevant to your study. If you are not sure if a list item applies to your research, read the appropriate section before selecting a response.

Materials & experimental systems

n/a	Involved in the study
☒	☐ Antibodies
☒	☐ Eukaryotic cell lines
☒	☐ Palaeontology and archaeology
☒	☐ Animals and other organisms
☒	☐ Clinical data
☒	☐ Dual use research of concern

Methods

n/a	Involved in the study
☒	☐ ChIP-seq
☒	☐ Flow cytometry
☒	☐ MRI-based neuroimaging
